# Evidence of the Zoonotic Transmission of *Cryptosporidium* among Children and Pets

**DOI:** 10.3390/pathogens12121393

**Published:** 2023-11-27

**Authors:** Natalia Marinho Dourado Coelho, Willian Marinho Dourado Coelho, Jancarlo Ferreira Gomes, Marcelo Vasconcelos Meireles, Walter Bertequini Nagata, Valéria Marçal Felix de Lima, Thais Rabelo Santos-Doni, Vitória Beatriz Silva, Luiz da Silveira Neto, Alex Akira Nakamura, Katia Denise Saraiva Bresciani

**Affiliations:** 1 Faculdade de Medicina Veterinária, Universidade Estadual Paulista (Unesp), Araçatuba 16050-680, São Paulo, Brazil; nataliamdcoelho2020@gmail.com (N.M.D.C.); m.meireles@unesp.br (M.V.M.); valeria.lima@unesp.br (V.M.F.d.L.); alex.nakamura@unesp.br (A.A.N.); 2 Faculdade de Ciências Agrárias, Fundação Educacional de Andradina, Andradina 16901-160, São Paulo, Brazil; willianmarinho@hotmail.com; 3 Faculdade de Ciências Médicas e Instituto de Computação, Universidade Estadual de Campinas (UNICAMP), Campinas 13083-887, São Paulo, Brazil; jgomes@ic.unicamp.br; 4 Escritório de Defesa Agropecuária, Coordenadoria de Defesa Agropecuária, Secretaria de Agricultura e Abastecimento do Estado de São Paulo, Lins 16400-050, São Paulo, Brazil; walter.bn@hotmail.com; 5 Instituto de Ciências Agrárias, Universidade Federal do Vale do Jequitinhonha e Mucuri (UFVJM), Unaí 38610-000, Minas Gerais, Brazil; rabelo.vet@hotmail.com; 6 Imunologia e Vacinologia, Curso de Engenharia de Bioprocessos e Biotecnologia, Universidade Federal do Tocantins (UFT), Gurupi 77410-530, Tocantins, Brazil; vitoria.beatriz@mail.uft.edu.br (V.B.S.); luiz.silveira@uft.edu.br (L.d.S.N.)

**Keywords:** animals, cats, cryptosporidiosis, dogs, epidemiology, One Health, pets, prevalence, risk factors, zoonosis

## Abstract

We investigated the zoonotic transmission of *Cryptosporidium* among the children (*n* = 188), dogs (*n* = 133), and cats (*n* = 55) living in 188 households. Fecal samples were examined using ELISA and confirmed via nested PCR. Coproantigens oocysts were detected in 3.7% of children, 8.3% of dogs, and 5.5% of cats. We found strong evidence of two cases of the zoonotic transmission of *Cryptosporidium canis* between children and dogs. Furthermore, four children and their respective pets (one dog and three cats) were infected with *Cryptosporidium parvum*, but we cannot exclude the hypotheses that the oocysts were transmitted from children to animals or that both hosts were infected by a shared source, such as contaminated water or food. The presence of an infected animal elevated the risk of zoonotic transmission by 129.7-fold (95% CI: 13.92–1209.68). Furthermore, sharing a bed with pets was identified as a risk factor for infection in children (OR: 9.9, 95% CI: 1.37–71.2). In conclusion, the zoonotic transmission of *Cryptosporidium* among children and pets cohabiting in the same household may be quite common, especially when infected animals lie or sleep on children’s beds. These findings unequivocally highlight the public health concern surrounding *C. canis*.

## 1. Introduction

*Cryptosporidium* is a genus of protozoa known to cause gastroenteritis in both humans and animals. Infection typically occurs through the ingestion of oocysts found in the contaminated water and food sources in direct contact with infected individuals. The most prominent clinical symptom of cryptosporidiosis is diarrhea, which tends to be self-limiting in immunocompetent individuals but can become chronic and potentially life-threatening for immunosuppressed patients [1,2]. As a result, this pathogen garnered increased attention during the AIDS epidemic in the 1980s [3].

In regions such as Africa and Asia, *Cryptosporidium* is the second most important causative agent of diarrheic disease in children under 5 years and one of the major causes of mortality from infectious disease in toddlers aged 12–23 months in developing countries [4]. The primary symptoms observed in children include watery diarrhea, abdominal pain, fatigue, nausea, fever, vomiting, headache, and eye pain [5].

Currently, over 40 species and more than 120 genotypes of *Cryptosporidium* have been identified [6]. *Cryptosporidium hominis* and *C. parvum* are responsible for over 90% of human infections, while C. *felis* and *C. canis*, species typically associated with cats and dogs, have also been reported [7]. Conventional microscopy and immunodiagnostic assays cannot differentiate between *Cryptosporidium* oocysts due to their high morphological similarity and the presence of conserved antigens [8,9]. Therefore, the use of molecular characterization is crucial to provide new insights into the epidemiology of cryptosporidiosis, including the potential risk of transmission among humans and companion animals.

There are few reports on the zoonotic transmission of *Cryptosporidium* among children and dogs and cats in households [7,10,11,12], but it is unclear whether these pets play a minor role on the epidemiology of cryptosporidiosis or if this low occurrence is underestimated because it is still under-investigated. Hence, we investigated the zoonotic transmission of *Cryptosporidium* species among children and dogs and cats living in the same households, and analyzed the risk factors of infection. 

## 2. Materials and Methods

### 2.1. Sampling

Stool samples were collected from 188 children, 133 dogs, and 55 cats residing in 188 households in the municipality of Andradina, São Paulo State, Brazil. The following inclusion criteria were applied for sample selection: each household contained only one child aged zero to 10 years and one dog or one cat, which was considered the sampling unit. Families with more than one child, children older than 10 years, or with more than one pet were excluded from the investigation.

Fecal samples were divided into three aliquots: 1 g was preserved in 10% formalin for direct ELISA, while two portions of 200 μg each were frozen at −20 °C in DNAse and RNAse-free microcentrifuge tubes for nested PCR (nPCR) analysis. Stool consistency was classified as diarrheic (liquid or pasty) or non-diarrheic (firm) according to the Bristol Stool Form Scale [13]. Additionally, parents completed a structured questionnaire to investigate potential risk factors. 

### 2.2. Laboratory Techniques

All stool samples were tested for the presence of *Cryptosporidium* oocysts’ antigen using the *Cryptosporidium* II™ commercial kit (TechLab, Blacksburg, VA, USA) [14]. Briefly, 100 μL of diluent was added to each well of the microplate. Subsequently, 50 μL of each fecal sample preserved in 10% buffered formalin was transferred to individual wells. After incubation for one hour, the microplate was washed four times. Then, 50 μL of conjugated antibody was added to each well. Following a 30 min incubation, the plate was washed four times. A volume of 100 μL of substrate was added to each well. After a 10 min incubation, 50 μL per well of stop solution was added. Two minutes later, the reactions were read using an ELISA plate reader (Spectra Count, Packard Bio Science Company, Meriden, CT, USA) at 490 nm. Samples were considered positive if the optical density was ≥0.150 [15]. Only fecal samples from children that tested positive via the ELISA and from dogs or cats that had contact with an infected child in the same household were submitted to nPCR. Fecal samples from dogs or cats living with non-reactive children were not subjected to molecular analyses, because our objective was to investigate zoonotic transmission.

Genomic DNA was extracted from frozen fecal samples (−20 °C) from children, dogs, and cats, using the QIAamp^®^ DNA Stool Mini Kit (Qiagen, Germantown, MD, USA). Subsequently, for the amplification of a fragment of the 18S rRNA gene, nPCR was performed with the primers 5′ TTC TAG AGC TAA TAC ATG CG 3′ and 5′ CCC ATT TCC TTC ACA GAA GGA 3′ for the primary reaction (amplicon size 1325 bp), and primers 5′ GGA AGG GTT GTA TTT ATT AGA TAA AG 3′ and 5′ AAG GAG TAA GGA ACA ACC TCC to 3′ for the secondary reaction (amplicon size 826–840 bp) [16]. All second-step PCR products were identified via electrophoresis on a 1.5% agarose gel, stained with GelRed^®^ (Biotium, Fremont, CA, USA), and visualized with an ultraviolet light transilluminator. Ultrapure water was used as a negative control, and DNA from *C. galli* [EU543270] and *C*. *parvum* [GQ227477] served as positive controls for nPCR reactions.

Following the secondary PCR reaction, DNA fragments were purified using the QIAquick Gel Extraction kit (Qiagen) and sequenced using the ABI Prism Dye Terminator Cycling Sequencing kit (Applied Biosystems, Foster City, CA, USA) in an automated ABI 3730XL sequencer (Applied Biosystems, Foster City, CA, USA). Amplicons were sequenced in both directions using the nested primers. The consensus sequence was determined using Codoncode Aligner version 4.0.1 (CodonCode Corporation, Dedham, MA, USA). Consensus sequences were aligned to homologous sequences downloaded from Genbank using ClustalW [17] and the BioEdit sequence alignment editor [18].

### 2.3. Statistical Analyses

Statistical analyses were performed using the STATA/SE Software, Version 16.1 (Stata Corp LLC, College Station, TX, USA). The statistical significance level was set at ≤0.05.

For inferential statistics, the detection of *Cryptosporidium* coproantigens was considered the dependent variable, and the following variables were considered as explanatory or independent: age (children: ≤24, 25 > 59, ≥60 months of age; dog/cat: up to one year, two to seven years, over seven years), sex (male or female), consistency of feces (diarrheic or not diarrheic), animal (cat or dog), does your child wash their hands after playing with the pet? (yes or no), does the animal (dog and/or cat) usually lick the face and mouth of the child(ren)? (yes or no), do children play with sand at home or at school? (yes or no), does your child wash their hands after using the bathroom? (yes or no), do you have a water and sewage system? (yes or no), does the dog or cat usually lie down or sleep in bed with the child? (yes or no), where the child drinks water from (filtered or faucet), does the animal have street access? (yes or no), and is the animal dewormed? (yes or no).

Chi-square or Fisher’s exact tests were employed to assess the statistical significance of the association between the variables. To explore the independent risk factors for each explanatory variable, all variables demonstrating a *p*-value of ≤0.25 in univariate analysis were subjected to multivariate logistic regression [19]. Utilizing an initial screening *p*-value cut-off point of 0.25 is recommended, as the conventional probability of 0.05 might overlook variables that are known to be significant. The probability ratio (odds ratio, OR) and the corresponding 95% confidence intervals (95% CI) were calculated using both univariate and multiple logistic regression models [20]. 

## 3. Results

*Cryptosporidium* coproantigens were detected in 3.7% (7/188) of children, in 5.7% (3/55) of cats, and 8.3% (11/133) of dogs (Table 1). In children, cats, and dogs that exhibited the symptom of diarrhea, the prevalence rates were 16.3% (7/43), 27.3% (3/11), and 25.0% (8/32), respectively.

The results of the univariable and multivariable regression models exploring the risk factors in children are presented in Table 2. A multivariable logistic regression model of the predictors of *Cryptosporidium* was performed on all variables (the age group of 25 to 59 months, diarrhea, the lack of an installed water and sewage system in the household, and sleeping or lying with the animal) that showed a *p* value ≤ 0.25 in the univariate analysis.

Overall, the predictor that remained independently associated with *Cryptosporidium* in the multivariate logistic regression model was sleeping or lying with the animal (adjusted odds ratio [aOR]: 9.9, 95% CI: 1.37–71.2).

There was no association with other variables investigated. Regarding hygiene habits, most children did not wash their hands after using the toilet or playing with their animals and allowed animals to lick their mouth and face. Almost half of the children drank tap water and lived in homes without access to water and sewage systems.

The prevalence of *Cryptosporidium* in dogs was 8.3% (11/133; 95% CI: 3.6–13.0). Applying the multivariate logistic regression model, tests of the association between the *Cryptosporidium* infection in dogs and potential predictors showed that diarrhea was a significant (*p* < 0.001) independent predictor for the infection. Among the 11 positive animals, eight (72.7%) presented with diarrhea. Associations between the infection and the age range of 2 to 7 years (*p* = 0.208) and access to streets (*p* = 0.0037) were found in the univariate analysis (Table 3). 

In cats, *Cryptosporidium* coproantigens were detected in 5.5% (3/55; 95% CI: 0–11.5) of samples. The infection appears to have contributed to the occurrence of diarrhea in the feline host (*p* = 0.006). No evidence of other infection risk factors in dogs and cats was found (Table 3).

Among the seven infected children, six (85.7%) lived with an animal that was also positive. Of the 11 infected dogs, only three (27.3%) lived with infected children. Samples from the eight dogs living with non-reactive children were not subjected to molecular analysis, because our objective was to investigate the zoonotic transmission of the parasite. *C. parvum* was identified in five children, one dog, and three cats, while two children and their respective dogs were infected with *C. canis* (Table 4). These species shared 100% similarity to MF589922 and EU754833, respectively. 

In the present study, children living with a pet (cat or dog) were 129.75 times more likely to have a *Cryptosporidium* infection (Table 5).

## 4. Discussion

Our main objective was to investigate the zoonotic transmission and risk factors of *Cryptosporidium* between children under 10 years old and dogs/cats in the same household. Coproantigens’ oocysts detection and nPCR were used as the screening and confirmatory techniques, respectively. Molecular characterization was performed through the sequencing of amplified 18S rRNA gene fragments.

We found strong evidence of the zoonotic transmission of *C. canis* in two children and their dogs, given that dogs serve as the primary host for this protozoan species [21]. Furthermore, four children and their respective pets (one dog and three cats) were infected with *C. parvum*. This species includes over 20 gp60 subtype families. Typically, human infections are associated with zoonotic subtype families IIa and IId, whereas subtype IIc is exclusively linked to anthropogenic transmission [21]. Although the gp60 gene sequences were not analyzed in our study, it is improbable that the four children and their pets were infected with different gp60 subtype families simultaneously within the same household. However, we did not exclude the possibility that *C. parvum* oocysts were transmitted from children to animals, or that both hosts were infected through a shared source, such as contaminated water or food.

Although *C. felis* and *C. canis* are the fourth and fifth most common *Cryptosporidium* species in humans, few studies have investigated the infection of humans and pets living in the same household [21]. Zoonotic transmissions of *C. canis* and *C. parvum* between children and dogs have been reported in Peru [10] and Egypt [11], respectively. In adults, cats have served as a source of *C. parvum* infection in Chile [22], and *C. felis* in Sweden [23]. These two cases of zoonotic transmission with *C. canis* and *C. felis* have been recently confirmed by subtype analyses, with identical subtypes being found in both humans and their pets [24,25]. In these studies, the patients involved included children and immunocompetent adults, suggesting that these pet pathogens are threats to both immunocompromised and immunocompetent persons. Curiously, the *C. felis* species was not found in our study.

In the univariate analysis, the presence of *Cryptosporidium* coproantigens was associated with the occurrence of diarrhea in children (*p* < 0.001), but this relationship was not confirmed via the multivariate analysis, which suggests the influence of multiple risk factors. It is important to note that, in the major cases of the zoonotic transmission of *Cryptosporidium* confirmed within households, both interacting hosts were asymptomatic [11,22] or only the humans presented with diarrhea [10]. The clinical manifestation of cryptosporidiosis in humans and pets living together and at the same time is rare [23]. Of the seven children infected by the parasite, six (85.7%) did not wash their hands after using the bathroom, five (71.4%) did not have a water and sewage system installed in their residence, and three (42.9%) drank water from the tap. All of these variables can serve as confounding predictors for the occurrence of diarrhea, as they favor the occurrence of gastrointestinal disorders caused by other pathogens frequently transmitted by contaminated water and food, such as *Blastocystis hominis* [26,27], *Giardia duodenalis* [26,28,29], *Escherichia coli* enteropathogenic [29,30], *Shigella* spp. [30], *Salmonella* spp. [30], *Yersinia* spp. [30], Astrovirus [29], and Rotavírus [31].

Most published epidemiological surveys typically focus on examining fecal samples from a single host species within a household. Frequently, these studies do not investigate the occurrence of the parasite in other interacting host species, particularly when they are asymptomatic. Indeed, the source of infection is often attributed to contaminated water and food, because cryptosporidiosis is traditionally classified as a foodborne and waterborne disease [26,32,33,34,35,36,37,38]. This leads us to believe that the transmission of *Cryptosporidium* among humans and their pets is underestimated, because the earlier conclusion might hide the real problem: a zoonotic transmission. 

Some investigations suggest that contact with animals increases the likelihood of *Cryptosporidium* infection in humans [39,40,41,42,43], but it remains controversial [26,32,33,44,45]. It is important to note that fecal samples from humans, dogs, and cats living in the same household are rarely analyzed in cross-sectional studies [40,45]. Typically, previous conclusions have been based on comparing the results obtained via diagnostic techniques to structured questionnaire data [26,32,39,43] and/or considered the presence of *C. canis* and *C. felis* as an indicator of low zoonotic transmission risk [34,46]. These extrapolations from earlier findings might have biased previous conclusions. This context highlights a crucial question: are children and pets in the same household sufficient to represent a risk factor? What is the chance of zoonotic transmission occurring when children cohabitate with an infected pet, and under what circumstances?

While children and pets cohabiting in the same household alone did not contribute significantly to *Cryptosporidium* infection (*p* = 0.419), the presence of an infected animal increased the risk of zoonotic transmission by 129.7 times (*p* < 0.00001). These results underscore the likelihood of zoonotic transmission. We believe that the animals might have carried the oocysts to the children’s beds, considering that this habit was the only risk factor confirmed via multivariate analysis. 

It is important to highlight a similar study conducted in the province of Álava, Northern Spain, where 63 families with children, dogs, and cats living together provided individual fecal samples from each member of the household, including pets. *Cryptosporidium* spp. oocysts were found in a single household in which a cat and its owner were infected, but zoonotic transmission was not confirmed molecularly. Unfortunately, the risk factor analysis was limited due to the low number of positive samples [45]. Some methodological differences may explain the divergence in our results. All human fecal samples examined in our study were collected from children under 10 years of age, who are more susceptible to *Cryptosporidium* infection. Environmental, infrastructural, and behavioral factors can also influence the occurrence of the disease [47].

The occurrence of *Cryptosporidium* to the order of the 3.7% that was found in the fecal samples from children was expected for an upper middle-income country, where the prevalence varies from 5.0 to 16.2% [47]. However, the results found in this research are below those of most epidemiological surveys conducted in Brazil [26,28,29,32,33,35,36,37,38,44]. This divergence may be related to differences in the sampling methodologies and diagnostic techniques used in these studies.

In Brazil, most epidemiological surveys of *Cryptosporidium* in children use non-probability sampling methods, in addition to adopting acute/persistent diarrhea [27,38,44,48], children attending daycare centers [26,32,36], or communities with precarious basic sanitation [33,35], as inclusion criteria.

In dogs, *Cryptosporidium* oocyst antigens were detected in 8.3% of fecal samples, similar to other epidemiological surveys conducted in Brazil [49,50,51], where the occurrence of the parasite in this host ranged from below 3.0% [52,53,54,55] to above 25% [31,56,57]. Worldwide, the expected prevalence of *Cryptosporidium* in dogs is approximately 10% [58]. The presence of oocysts in feces contributed to the occurrence of diarrhea (*p* = 0.004), confirming the clinical importance of *Cryptosporidium* infection for the canine host. 

In Brazil, the occurrence of *Cryptosporidium* in cats varies from 0.5% [59] to 30% [60]. The results of this study are in line with most epidemiological surveys in Brazil [31,57,61,62,63,64,65,66,67,68]. The clinical relevance of *Cryptosporidium* in the feline host remains uncertain [69,70]. We found an association between the presence of *Cryptosporidium* oocysts and the occurrence of diarrhea (*p* = 0.006), but the low number of positive samples reduced the reliability of the results of the uni- and multivariate analyses.

The sensitivity and specificity values of the diagnostic techniques used in this study were not calculated because the samples were not examined in a paired manner. Coproantigens’ oocyst detection and nPCR were employed as screening and confirmatory techniques, respectively. However, all positive results via direct ELISA were confirmed via nPCR, followed by genetic sequencing.

In conclusion, our research suggests that the zoonotic transmission of *Cryptosporidium* among children under 10 years old and the dogs and cats living in the same household may be quite common, especially when infected animals lie or sleep on children’s beds. These findings unequivocally highlight the public health concern surrounding *C. canis*. Under conducive circumstances, transmission from dogs to humans is possible. As a result, we recommend that children do not share their beds with pets, particularly under immunosuppression conditions.

## Figures and Tables

**Table 1:** Detection of *Cryptosporidium* sp., via ELISA, in children and their respective pets (cat/dogs).

Species/Sex	ELISA		Total
	Negative *n* (%)	Positive *n* (%)	
**Dogs**			
Female	39 (90.7)	4 (9.3)	43
Male	83 (92.2)	7 (7.8)	90
TOTAL	122 (92.2)	11 (8.3)	133
**Cats**			
Female	24 (96.0)	1 (4.0)	25
Male	28 (93.3)	2 (6.7)	30
TOTAL	52 (94.6)	3 (5.5)	55
**Children**			
Female	85 (97.7)	2 (2.3)	87
Male	96 (95.1)	5 (4.9)	101
TOTAL	181 (96.3)	7 (3.7)	188

**Table 2:** Univariable analysis and multivariable logistic regression model of the presence of *Cryptosporidium* coproantigens in children.

Risk Factors	*Cryptosporidium* Coproantigens (ELISA)			Univariable Analysis		Multivariable Logistic Regression	
	Negative *n* (%)	Positive *n* (%)	Total	OR (95% CI) ^1^	*p* ^2^	AOR (95% CI) ^3^	*p* ^2^
Sex							
Female	85 (97.7)	2 (2.3)	87 (46.3)		0.454		
Male	96 (95.1)	5 (4.9)	101 (53.7)	2.21 (0.42 < OR < 11.71)			
Age (months)							
≤24	53 (96.4)	2 (3.6)	55 (29.3)	1.03 (0.20 < OR < 5.50)	0.968		
25 > 59	81 (94.2)	5 (5.8)	86 (45.7)	3.08 (0.58 < OR < 16.32)	0.185 *	0.96 (0.13 < OR < 7.12)	0.973
≥60	47 (100)	0 (0)	47 (25.0)	NC	0.992		
Fecal consistency							
Not Diarrhea	145 (100)	0 (0)	145 (77.1)	NC	<0.001 *	1	0.997
Diarrhea	36 (83.7)	7 (16.3)	43 (22.9)				
Living with animal							
Cat	52 (94.6)	3 (5.4)	55 (29.3)	1.86 (0.40 < OR < 8.60)	0.419		
Dog	129 (96.3)	4 (3.0)	133 (70.7)				
Washing hands after playing with the pet							
No	172 (96.1)	7 (3.9)	179 (95.2)	NC	1.00		
Yes	9 (100)	0 (0)	9 (4.8)				
Does the animal (dog and/or cat) usually lick the face and mouth of the child(ren)?							
No	28 (93.3)	2 (6.7)	30 (16.0)	2.18 (0.40 < OR < 11.83)	0.364		
Yes	153 (96.8)	5 (3.2)	158 (84.0)				
Playing with sand at home or at school							
No	25 (92.6)	2 (7.4)	27 (14.4)	2.50 (0.46 < OR < 13.57)	0.290		
Yes	156 (96.9)	5 (3.1)	161 (85.6)				
Washing hands after using the toilet							
No	146 (96.1)	6 (3.9)	152 (80.9)	1.44 (0.17 < OR < 12.14)	0.740		
Yes	35 (97.2)	1 (2.8)	36 (19.1)				
Access to a water and sewage system							
No	80 (94.1)	5 (5.8)	85 (45.2)	3.16 (0.60 < OR < 16.70)	0.176 *	5.09 (0.69 < OR < 37.16)	0.109
Yes	101 (98.1)	2 (1.9)	103 (54.8)				
Lying down or sleeping in bed with the pet							
No	142 (98.6)	2 (1.4)	144 (76.0)				
Yes	39 (88.6)	5 (11.4)	44 (24.0)	9.10 (1.70 < OR < 48.73)	0.006 *	9.88 (1.37 < OR < 71.20)	0.023
Source of water							
Filtered or boiled	90 (96.8)	3 (3.2)	93 (49.5)				
Tap	91 (95.8)	4 (4.2)	95 (50.5)	1.32 (0.29 < OR < 6.06)	0.722		

^1^ OR: odds ratio; reference group marked as OR = 1; CI: confidence interval; ^2^ Pearson’s chi-square; ^3^ AOR: adjusted odds ratio; NC: not calculated; * significant association (*p* < 0.25).

**Table 3:** Univariable analysis and multivariable logistic regression model of the presence of *Cryptosporidium* coproantigens in pets (cat/dogs).

Pet	Risk Factors	*Cryptosporidium* Coproantigens (ELISA)			Univariable Analysis *		Multivariable Logistic Regression	
		Negative *n* (%)	Positive *n* (%)	Total	OR (95% CI) ^1^	*p* ^2^	AOR (95% CI ) ^3^	*p* ^2^
Cat	Sex							
	Female	24 (96.0)	4 (4.0)	25 (45.5)		0.668		
	Male	28 (93.3)	2 (6.7)	30 (54.5)	1.71 (0.15 < OR < 20.10)			
	Age (year)							
	≤1	33 (91.7)	3 (8.3)	36 (65.5)	NC	0.544		
	2 ≥ 7	19 (100)	0 (0)	19 (34.5)	NC	0.544		
	>7	0 (0)	0 (0)	0 (0)	NC			
	Fecal consistency							
	Not Diarrhea	44 (100)	0 (0)	44 (80)	NC	0.006		
	Diarrhea	8 (72.7)	3 (27.3)	11 (20)				
	Access to street							
	No	2 (100)	0 (0)	2 (3.6)	NC	NC		
	Yes	50 (94.3)	3 (5.7)	53 (96.4)				
	Dewormed							
	No	36 (92.3)	3 (7.7)	39 (70.9)	NC	0.996		
	Yes	16 (100)	0 (0)	16 (29.1)				
Dog	Sex							
	Female	39 (90.7)	4 (9.3)	43 (32.3)	1.21 (0.34 < OR < 4.40)	0.766		
	Male	83 (92.2)	7 (7.8)	90 (67.7)				
	Age (year)							
	≤1	58 (89.2)	7 (10.8)	65 (48.9)	1.93 (0.54 < OR < 6.93)	0.358		
	2 ≥ 7	58 (95.1)	3 (4.9)	61 (45.9)	0.41 (0.11 < OR < 1.63)	0.208	0.23 (0.05 < OR < 1.10)	0.065
	>7	6 (85.7)	1 (14.3)	7 (5.2)	1.93 (0.21 < OR < 17.68)	0.559		
	Fecal consistency							
	Not Diarrhea	98 (97.0)	3 (3.0)	101 (75.9)		0.001		0.004
	Diarrhea	24 (75.0)	8 (25.0)	32 (24.1)	10.89 (2.69 < OR < 44.16)		11.76 (2.22 < OR < 62.41)	
	Access to street							
	No	51 (100)	0 (0)	51 (38.4)	NC	0.007		0.989
	Yes	71 (86.7)	11 (13.3)	82 (61.6)				
	Dewormed							
	No	73 (89.0)	9 (11.0)	82 (61.6)	3.02 (0.63 < OR < 14.58)	0.169	1.00 (0.15 < OR < 6.67)	0.990
	Yes	49 (96.1)	2 (3.9)	51 (38.4)				

^1^ OR: odds ratio. Reference group marked as OR = 1; CI: confidence interval. ^2^ Pearson’s chi-square. ^3^ NC: not calculated. * Significant association (*p* < 0.25).

**Table 4:** Molecular characterization of *Cryptosporidium* in feces from children and their respective pets (cat/dogs).

Household	Children		Animal		
	Molecular Characterization	Diarrhea	Pet	Molecular Characterization	Diarrhea
1	*C. canis*	Yes	Dog	*C. canis*	Yes
2	*C. canis*	Yes	Dog	*C. canis*	Yes
3	*C. parvum*	Yes	Dog	*C. parvum*	Yes
4	*C. parvum*	Yes	Dog	Not amplified	No
5	*C. parvum*	Yes	Cat	*C. parvum*	Yes
6	*C. parvum*	Yes	Cat	*C. parvum*	Yes
7	*C. parvum*	Yes	Cat	*C. parvum*	Yes

**Table 5:** Univariable analysis model of the presence of *Cryptosporidium* sp. in children and their respective pets (cat/dogs).

	Pets	Negative *n* (%)	Positive *n* (%)	Total	OR (CI 95%) ^1^	*p* ^2^
Children						
Negative		173 (95.6)	8 (4.4)	181 (96.3)	129.75 (13.92 < OR < 1209.68)	< 0.00001
Positive		1 (14.3)	6 (85.7)	7 (3.7)		

^1^ OR: odds ratio. Reference group marked as OR = 1; CI: confidence interval. ^2^ Pearson’s chi-square (*p* < 0.05).

## Data Availability

The data presented in this study are available within the article.
